# Absolute and Relative Risks of Kidney Outcomes Associated With Lithium vs Valproate Use in Sweden

**DOI:** 10.1001/jamanetworkopen.2023.22056

**Published:** 2023-07-07

**Authors:** Alessandro Bosi, Catherine M. Clase, Laura Ceriani, Arvid Sjölander, Edouard L. Fu, Björn Runesson, Zheng Chang, Mikael Landén, Rino Bellocco, Carl-Gustaf Elinder, Juan Jesus Carrero

**Affiliations:** 1 Department of Medical Epidemiology and Biostatistics, Karolinska Institutet, Stockholm, Sweden; 2 Department of Medicine and Health Research Methods, Evidence, and Impact, McMaster University, Hamilton, Ontario, Canada; 3 Department of Statistics and Quantitative Methods, University of Milano-Bicocca, Milan, Italy; 4 Division of Pharmacoepidemiology and Pharmacoeconomics, Department of Medicine, Brigham and Women’s Hospital and Harvard Medical School, Boston, Massachusetts; 5 Department of Public Health and Clinical Medicine, Umeå University, Umeå, Sweden; 6 Department of Neuroscience and Physiology, Gothenburg University, Gothenburg, Sweden; 7 Renal Medicine, Department of Clinical Science, Intervention, and Technology, Karolinska Institutet, Stockholm, Sweden; 8 Division of Nephrology, Department of Clinical Sciences, Karolinska Institutet, Danderyd Hospital, Stockholm, Sweden

## Abstract

**Importance:**

Among patients with bipolar disorder, discordant findings have been published on the nephrotoxic effects of lithium therapy.

**Objective:**

To quantify absolute and relative risks of chronic kidney disease (CKD) progression and acute kidney injury (AKI) in people who initiated lithium compared with valproate therapy and to investigate the association between cumulative use and elevated lithium levels and kidney outcomes.

**Design, Setting, and Participants:**

This cohort study had a new-user active-comparator design and used inverse probability of treatment weights to minimize confounding. Included patients initiated therapy with lithium or valproate from January 1, 2007, to December 31, 2018, and had a median follow-up of 4.5 years (IQR, 1.9-8.0 years). Data analysis began in September 2021, using routine health care data from the period 2006 to 2019 from the Stockholm Creatinine Measurements project, a recurrent health care use cohort of all adult residents in Stockholm, Sweden.

**Exposures:**

New use of lithium vs new use of valproate and high (>1.0 mmol/L) vs low serum lithium levels.

**Main Outcomes and Measures:**

Progression of CKD (composite of >30% decrease relative to baseline estimated glomerular filtration rate [eGFR] and kidney failure), AKI (by diagnosis or transient creatinine elevations), new albuminuria, and annual eGFR decrease. Outcomes by attained lithium levels were also compared in lithium users.

**Results:**

The study included 10 946 people (median [IQR] age, 45 [32-59] years; 6227 female [56.9%]), of whom 5308 initiated lithium therapy and 5638 valproate therapy. During follow-up, 421 CKD progression events and 770 AKI events were identified. Compared with patients who received valproate, those who received lithium did not have increased risk of CKD (hazard ratio [HR], 1.11 [95% CI, 0.86-1.45]) or AKI (HR, 0.88 [95% CI, 0.70-1.10]). Absolute 10-year CKD risks were low and similar: 8.4% in the lithium group and 8.2% in the valproate group. No difference in the risk of developing albuminuria or the annual rate of eGFR decrease was found between groups. Among more than 35 000 routine lithium tests, only 3% of results were in the toxic range (>1.0 mmol/L). Lithium values greater than 1.0 mmol/L, compared with lithium values of 1.0 mmol/L or less, were associated with increased risk of CKD progression (HR, 2.86; 95% CI, 0.97-8.45) and AKI (HR, 3.51; 95% CI, 1.41-8.76).

**Conclusions and Relevance:**

In this cohort study, compared with new use of valproate, new use of lithium was meaningfully associated with adverse kidney outcomes, with low absolute risks that did not differ between therapies. However, elevated serum lithium levels were associated with future kidney risks, particularly AKI, emphasizing the need for close monitoring and lithium dose adjustment.

## Key Points

**Question:**

How is lithium therapy, compared with valproate therapy, associated with kidney outcomes?

**Findings:**

In this cohort study of 10 946 patients followed up for up to 10 years in Sweden, no significant differences in relative and absolute risk of chronic kidney disease progression, albuminuria, or acute kidney injury (AKI) were found among patients who received lithium compared with those who received valproate. However, high levels of lithium were strongly associated with AKI risks.

**Meaning:**

Initiation of lithium therapy was not associated with risk of chronic kidney disease, albuminuria, or AKI, but the association between higher lithium levels and AKI calls for continuous patient monitoring and lithium dose adjustment to avoid toxic levels.

## Teaser

This cohort study evaluates the association between lithium vs valproate therapy and kidney outcomes, including chronic kidney disease progression or acute kidney injury, among patients in Sweden.

## Introduction

Lithium is the most effective prophylactic treatment for bipolar disorder^1^ and augments the effects of other drugs in treatment-resistant depression.^2^ Use of lithium is constrained by concern over its nephrotoxic effects, a potential chronic tubulointerstitial nephritis resulting in decreased glomerular filtration rate (GFR; lithium nephropathy). However, the underlying pathophysiologic mechanism is not fully understood.^3,4^ Despite more than 5 decades of debate on the benefits and harms of this medication, the absolute and relative risks of kidney damage remain poorly characterized.^5,6^

A meta-analysis of trials and observational studies resulted in heterogeneous and inconclusive estimates because of limitations of the original studies and differences in outcome ascertainment.^7^ Subsequent studies, mostly large-scale observational studies from routine care, have provided conflicting results, potentially attributable to lack of an active comparator,^8,9^ restriction to elderly individuals,^10,11^ and the lack of information on serum lithium levels.^5,12^ Studies have used administrative codes to identify chronic kidney disease (CKD),^8,11,13^ which have low sensitivity; lacked information on GFR,^13^ which is a key confounder; or used a single GFR value to define outcomes,^9,10,14^ which introduces misclassification. The potential risk of acute kidney injury (AKI) has been studied only in case series and single-center studies.^12^ We aimed to quantify the absolute and relative risks of clinically relevant CKD progression or AKI in those who initiated lithium vs valproate treatment and the impact of treatment duration and intensity. We also investigated the association between lithium levels and kidney outcomes.

## Methods

### Data Sources

For this cohort study, we analyzed the Stockholm Creatinine Measurements (SCREAM) database, a health care use cohort of all adult residents in Stockholm, Sweden.^15^ Stockholm is an administrative region that had a population of 2.3 million citizens in 2021, all receiving universal health care within a unified health system. Administrative databases with complete information on demographic data, health care use, diagnoses and therapeutic surgical procedures, vital status, routine laboratory tests, and dispensed prescriptions were linked and deidentified by the Swedish National Board of Health and Welfare. Because the study used deidentified data, the requirement of informed consent was waived by the regional ethics review board. The study followed the Strengthening the Reporting of Observational Studies in Epidemiology (STROBE) reporting guideline.^16^

### Initiation and Cumulative Use of Lithium or Valproate Therapy

We adopted a new-user active-comparator design comparing initiation of lithium therapy with initiation of valproate therapy, a drug with similar indications but no suspected nephrotoxicity. The study population consisted of all adults (aged ≥18 years) who newly initiated therapy with either drug between January 1, 2007, and December 31, 2018. We began the analysis in September 2021, using routine health care data from the period 2006 to 2019. New initiation was defined as a first registered pharmacy dispensation, with no previous dispensation of either drug since June 2005. The date of first dispensation was defined as baseline and start of follow-up. Patients were excluded if they had a history of kidney replacement therapy (KRT; maintenance dialysis or kidney transplantation).

In the primary analysis, we used an intention-to-treat design, in which patients were analyzed based on the first prescription of lithium or valproate, regardless of drug discontinuation, because the possible chronic toxic effects of lithium on kidney function may develop after many years or after stopping use of the medication. In a secondary analysis, we calculated the cumulative use of these drugs by collecting information on each subsequent dispensation over time. For any drug, defined daily dosages (DDDs) have been published: they are the mean maintenance dose per day when used for its main indications in adults.^17^ We quantified the overall amount of medication dispensed by calculating the number of DDDs per dispensation: number of pills contained in the package multiplied by the amount of active principle per pill (in milligrams) and divided by the DDD (also in milligrams). We calculated the cumulative use of both lithium and valproate and treated them as time-dependent exposures, summing at each dispensation the total DDDs obtained since initiation, allowing medication switches and interruptions. This design allows us to compare similar lengths of treatment and exposure (DDDs) for patients taking the 2 medications.

### Long-Term Maintenance Lithium Levels While Receiving Lithium Therapy

To study long-term use, for participants still using lithium at 1 year after initiation, we defined a new baseline at that time. We hypothesized that high lithium levels in the long term (ie, serum concentration) would be associated with higher risk of adverse kidney outcomes compared with long-term low levels. We estimated the long-term level as the median of all levels during the first year of therapy, and at each subsequent lithium level measurement, we recalculated the median level using data from the previous 12 months. We categorized the median lithium level as chronically high according to 3 different thresholds: (1) median lithium level greater 0.8 mmol/L, based on the upper limit of our reference laboratory and consistent with a previous study^5^; (2) median lithium level greater than 0.9 mmol/L; and (3) median lithium level greater than 1.0 mmol/L, a threshold previously associated with risk of AKI.^12^ We examined median lithium levels as both fixed (baseline) and time-varying covariates.

### Study Covariates

Study covariates included sociodemographic characteristics (age, sex, and highest attained education), laboratory measurements, comorbidities, ongoing medications, and health care use (outpatient and inpatient contacts in previous year, overall and psychiatry related). The GFR was calculated with the 2009 CKD-EPI (Chronic Kidney Disease Epidemiology Collaboration) equation^18^ without correction for race (it is not legal to collect information on race in Sweden), using routine isotope-dilution mass spectrometry traceable serum or plasma creatinine measurements. We defined CKD at baseline as an annual estimated GFR (eGFR) less than 60 mL/min/1.73 m^2^. Algorithms used to define study covariates are detailed in eFigure 1 and eTables 1 and 2 in Supplement 1.

### Study Outcomes

The primary outcome was CKD progression, defined as the composite of KRT or a sustained 30% or greater decrease in eGFR from baseline. The secondary outcomes were AKI, specified a priori and using clinical diagnoses, as well as inpatient and outpatient creatinine values. Two post hoc outcomes, new albuminuria and annual decrease in GFR, were added after peer review (detailed in the eMethods in Supplement 1). In each analysis, patients were followed up until the outcome of interest, death, moving out of the Stockholm region, or the end of follow-up (December 31, 2018), whichever occurred first.

### Statistical Analysis

Continuous variables are presented as means (SDs) or medians (IQRs), depending on the distribution, and categorical variables as numbers (percentages). Incidence rates per 1000 person-years with 95% CIs were computed for all outcomes. We regarded *P* ≤ .05 as statistically significant; all hypothesis tests were 2-tailed.

For the main analysis (initiation of lithium vs valproate therapy), we used propensity scores with inverse probability of treatment weighting to control for baseline confounding.^19,20^ Robust variance estimation was used to calculate CIs after weighting. Weighted Cox proportional hazards regression was used to estimate hazard ratios (HRs) and 95% CIs between lithium vs valproate therapy initiation and outcomes, with time since initiation as the time scale. Covariates that did not achieve balance after inverse probability of treatment weighting were included in the model as additional confounders. Weighted Kaplan-Meier curves were plotted to display the cumulative incidence of outcomes over the follow-up period. We used a linear mixed model with random intercept and slope to analyze the annual rate of change in eGFR.

As sensitivity analyses, first, we used the alternative weighting method of overlap weights,^19^ and second, we explored the risk of detection bias (ie, differential outcome ascertainment) arising from differences in the frequency of testing by comparing rates of creatinine testing during follow-up in each treatment group. We explored the association between the cumulative use of lithium or valproate (ie, the number of DDDs dispensed since the beginning of therapy) and kidney outcomes through multivariable Cox proportional hazards regression with time-varying exposures and time-fixed (at baseline) confounders. We compared the risks between the cumulative use of lithium vs valproate by calculating the ratio between the HR from the single models. Thus, this HR compares kidney risks between both therapies given the same time-dependent long-term use (ie, given the same amount of DDDs dispensed).

We used multivariable Cox proportional hazards regression to explore the association between long-term lithium levels and kidney outcomes, with time-varying exposures and time-fixed (ie, baseline) confounders. There was no missingness for any of the baseline study covariates, except for attained education, which was missing in 2%. We opted to give them a missing category. Analyses were performed using R software, version 4.0.5 (R Project for Statistical Computing).^21^

## Results

### Population Characteristics

During 2007 to 2018, a total of 16 645 adults started lithium or valproate therapy in the region of Stockholm. After exclusion criteria were applied, 10 946 individuals (median [IQR] age, 45 [32-59] years; 6227 female [56.9%] and 4719 male [43.1%]) were included, of whom 5308 initiated lithium therapy and 5638 valproate therapy (eFigure 2 in Supplement 1). Their baseline characteristics are given in Table 1. The annual median (IQR) eGFR was 99 (85-112) mL/min/1.73 m^2^, and 2% of those treated with lithium and 5% of those treated with valproate had an annual eGFR less than 60 mL/min/1.73 m^2^. The pattern of prescription did not vary greatly throughout the observation period (eFigure 3 in Supplement 1).

**Table 1.** Baseline Characteristics of Patients Initiating Lithium or Valproate Treatment Before and After Weighting^a^

Characteristic	Before weighting			After weighting		
	Valproate (n = 5638)	Lithium (n = 5308)	SMD	Valproate (n = 5919)	Lithium (n = 4855)	SMD
Age, median (IQR), y	49 (34-66)	41 (30-53)	0.46	44 (31-58)	43 (31-56)	0.125
Sex						
Female	2909 (52)	3318 (62)	0.22	3381 (57)	2833 (58)	0.025
Male	2729 (48)	1990 (38)		2539 (43)	2022 (42)	
Attained education						
Compulsory school	1397 (25)	740 (14)	0.36	1098 (19)	846 (17)	0.04
Secondary school	2302 (41)	2161 (41)		2425 (41)	2050 (42)	
University	1783 (32)	2359 (44)		2295 (39)	1892 (39)	
Missing	156 (3)	48 (1)		102 (2)	68 (1)	
eGFR, median (IQR), mL/min/1.73 m^2^	97 (82-111)	101 (88-113)	0.21	99 (86-112)	101 (88-112)	0.10
eGFR category						
<60 mL/min/1.73 m^2^	413 (7)	90 (2)	0.29	302 (5)	100 (2)	0.16
>60 mL/min/1.73 m^2^	5225 (93)	5218 (98)		5618 (95)	4756 (98)	
Comorbidities						
Bipolar disorder	1034 (18)	3019 (57)	0.87	2430 (41)	2033 (42)	0.02
Depression	2293 (41)	3822 (72)	0.67	3493 (59)	3039 (63)	0.07
Manic episode	207 (4)	353 (7)	0.14	354 (6)	312 (6)	0.02
Anxiety disorder	1882 (33)	2756 (52)	0.38	2615 (44)	2341 (48)	0.08
Mental disorders attributable to psychoactive substance use	1000 (18)	1022 (19)	0.05	1106 (19)	1014 (21)	0.06
Schizophrenia spectrum disorders	982 (17)	887 (17)	0.02	1003 (17)	925 (19)	0.06
Hyperthyroidism	76 (1)	55 (1)	0.03	69 (1)	52 (1)	0.008
Hypertension	1515 (27)	702 (13)	0.35	1162 (20)	887 (18)	0.04
Diabetes	593 (11)	265 (5)	0.21	447 (8)	411 (9)	0.03
Acute coronary syndrome	261 (5)	47 (1)	0.23	159 (3)	106 (2)	0.03
Other ischemic heart disease	458 (8)	103 (2)	0.29	290 (5)	181 (4)	0.06
Heart failure	359 (6)	47 (1)	0.30	210 (4)	136 (3)	0.04
Stroke	872 (16)	88 (2)	0.51	485 (8)	282 (6)	0.09
Other cerebrovascular disease	784 (14)	103 (2)	0.45	448 (8)	182 (4)	0.17
Atrial fibrillation	444 (8)	76 (1)	0.31	259 (4)	129 (3)	0.09
Arrhythmia	342 (6)	149 (3)	0.16	270 (5)	195 (4)	0.03
Peripheral vascular disease	168 (3)	50 (1)	0.15	112 (2)	93 (2)	0.002
Valve disorders	91 (2)	18 (0)	0.13	56 (1)	65 (1)	0.04
Liver disease	231 (4)	164 (3)	0.05	200 (3)	171 (4)	0.007
Cancer	439 (8)	96 (2)	0.28	266 (5)	219 (5)	0.001
Medications						
Lamotrigine	789 (14)	1586 (30)	0.39	1367 (23)	1192 (25)	0.03
Carbamazepine	542 (10)	63 (1)	0.38	308 (5)	184 (4)	0.07
First-generation antipsychotics	921 (16)	806 (15)	0.03	945 (16)	857 (18)	0.04
Second-generation antipsychotics	1877 (33)	3014 (57)	0.49	2755 (47)	2402 (50)	0.06
Other mood stabilizers	647 (12)	821 (16)	0.12	774 (13)	693 (14)	0.04
Antidepressants	2850 (51)	3907 (74)	0.49	3789 (64)	3220 (66)	0.05
Attention-deficit/hyperactivity disorder medication	369 (7)	424 (8)	0.06	431 (7)	453 (9)	0.07
Drugs used in addictive disorders	356 (6)	340 (6)	0.004	390 (7)	349 (7)	0.02
Opioids and pain medication	1362 (24)	963 (18)	0.15	1240 (21)	1081 (22)	0.03

Abbreviations: eGFR, estimated glomerular filtration rate; SMD, standardized mean difference.

^a^ Data are presented as number (percentage) of patients unless otherwise indicated. Weighting achieved through inverse probability of treatment weights. Additional characteristics used in the weighting model are given in eTable 3 in Supplement 1.

Patients who started lithium therapy were younger and more often female, with a higher prevalence of bipolar disorder, depression, and anxiety disorders than observed in those who started valproate therapy. Inverse probability of treatment weighting showed a good ability to balance measured confounders between both treatment groups, with most standardized mean differences less than 0.1 after weighting (Table 1; eTable 3 in Supplement 1).

### New Use of Lithium vs Valproate

During a median follow-up of 4.5 years (IQR 1.9-8.0 years), 182 individuals (3% of total) in the lithium group and 247 individuals (4% of total) in the valproate group experienced progression of CKD, corresponding to an incidence of 6.9 events per 1000 person-years in the lithium group and 8.9 events per 1000 person-years in the valproate group (Table 2). The median duration of lithium therapy was 4 years (IQR, 1.9-8.0 years), and the median duration of valproate therapy was 4 years (IQR, 1.8-8.0 years). Of the new users of lithium, 777 had at least 1 dispensation of valproate during follow-up; of the new users of valproate, 649 had at least 1 lithium dispensation.

**Table 2.** Number of Events, Incidence Rates, Absolute Risks, and Adjusted Hazard Ratios for the Association Between Initiation of Lithium vs Valproate Therapy and Kidney Outcomes^a^

	No. of events (IR per 1000 person-years)^b^	Follow-up, median (IQR), y	5-y Absolute risk (95% CI)	10-y Absolute risk (95% CI)	Adjusted HR (95% CI)
**CKD progression**					
Lithium	182 (6.9)	4.3 (1.9-7.8)	3.0 (2.6-3.7)	8.1 (6.7-9.6)	1.11 (0.86-1.45)
Valproate	247 (8.6)	4.4 (1.7-7.8)	3.3 (2.5-4.1)	8.2 (6.8-9.6)	1 [Reference]
**Acute kidney injury**					
Lithium	234 (9.0)	4.2 (1.8-7.8)	5.4 (4.3-6.5)	9.9 (8.3-11.3)	0.88 (0.70-1.10)
Valproate	536 (20.1)	4.2 (1.6-7.7)	7.2 (6.4-8.2)	13.1 (11.3-14.9)	1 [Reference]
**Albuminuria**					
Lithium	166 (6.9)	4.4 (2.0-8.0)	4.3 (2.9-6.4)	7.5 (5.7-9.9)	0.99 (0.74-1.33)
Valproate	272 (11.0)	4.6 (1.8-8.0)	4.9 (3.8-5.3)	8.1 (6.9-9.3)	1 [Reference]

Abbreviations: CKD, chronic kidney disease; HR, hazard ratio; IR, incidence rate.

^a^ Analyses were weighted for the following variables: age, sex, attained education, baseline estimated glomerular filtration rate, number of hospitalizations during previous year and psychiatry related, number of outpatient contacts during previous year and psychiatry related, number of outpatient contacts during previous year, pregnancy in the 2 years prior, total number of medications in the previous year, comorbidities (bipolar disorder, depression, manic episode, anxiety disorder, mental disorders attributable to psychoactive substance use, schizophrenia spectrum disorders, hypertension, diabetes, acute coronary syndrome, other ischemic heart disease, heart failure, stroke, other cerebrovascular diseases, valve disorders, atrial fibrillation, other arrhythmia, hyperthyroidism, cancer, and liver disease) and ongoing medications (lamotrigine, carbamazepine, first- and second-generation antipsychotic drugs, other mood stabilizers, antidepressants, attention-deficit/hyperactivity disorder medication, drugs used in addictive disorders, opioids and pain medications, antiepileptic drugs, β-blockers, calcium channel blockers, diuretics, angiotensin-converting enzyme inhibitors or angiotensin receptor blockers, lipid-lowering drugs, and nonsteroidal anti-inflammatory drugs).

^b^ Number of events (IRs) were calculated in the original, unweighted population.

**Figure 1.**
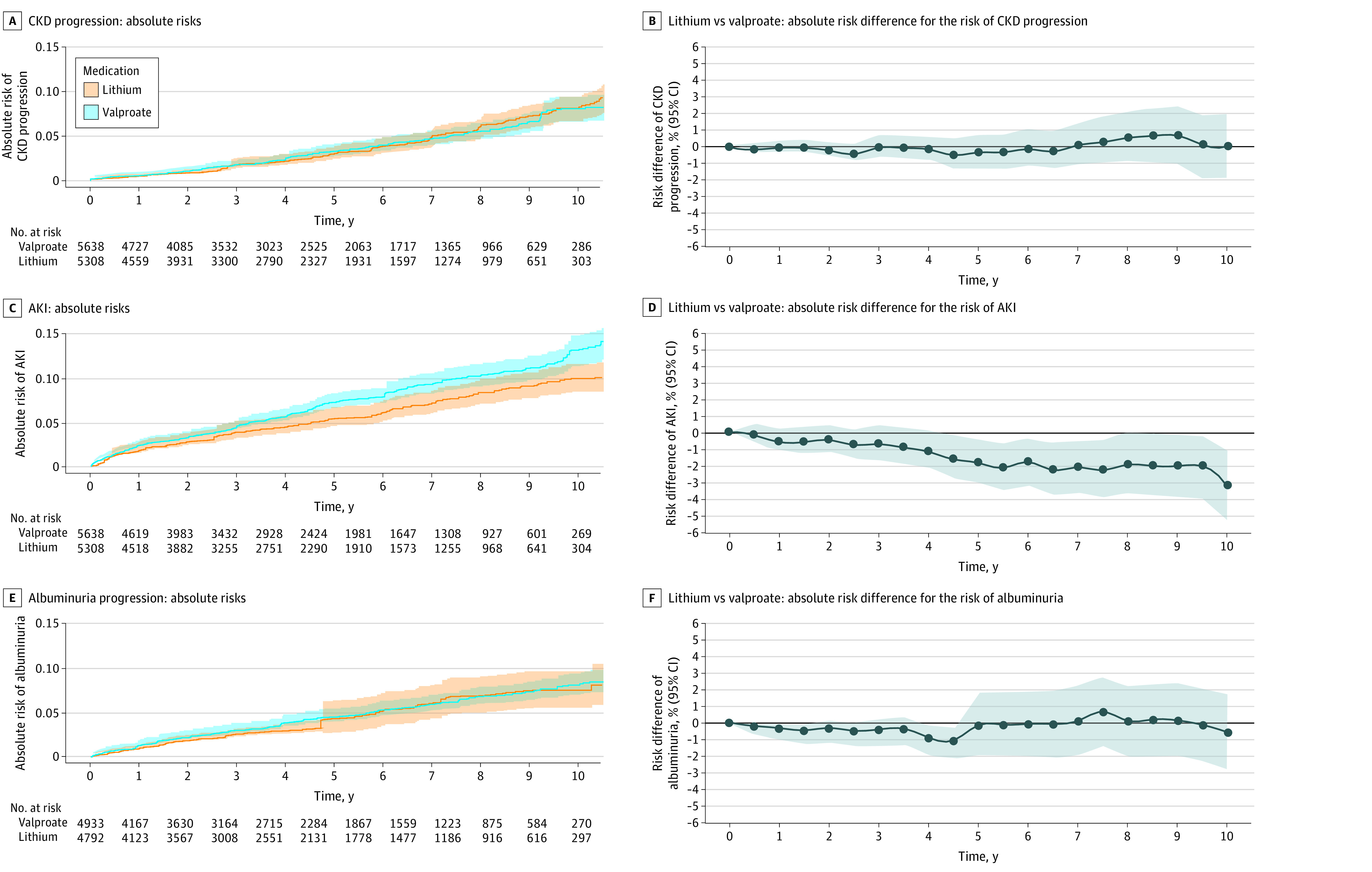
Weighted Cumulative Incidence Curves Showing the Risk of Chronic Kidney Disease (CKD) Progression, Acute Kidney Injury (AKI), and Albuminuria by Lithium or Valproate Initiation and Their Respective Absolute Risk Differences (Lithium vs Valproate) Throughout Follow-up Curves were weighted for the following covariates: age, sex, attained education, baseline estimated glomerular filtration rate, number of hospitalizations during previous year and psychiatry related, number of outpatient contacts during previous year and psychiatry related, number of outpatient contacts during previous year, pregnancy in the 2 years prior, total number of medications in the previous year, comorbidities (bipolar disorder, depression, manic episode, anxiety disorder, mental disorders attributable to psychoactive substance use, schizophrenia spectrum disorders, hypertension, diabetes, acute coronary syndrome, other ischemic heart disease, heart failure, stroke, other cerebrovascular diseases, valve disorders, atrial fibrillation, other arrhythmia, hyperthyroidism, cancer, and liver disease) and ongoing medications (lamotrigine, carbamazepine, first- and second-generation antipsychotic drugs, other mood stabilizers, antidepressants, attention-deficit/hyperactivity disorder medication, drugs used in addictive disorders, opioids and pain medications, antiepileptic drugs, β-blockers, calcium channel blockers, diuretics, angiotensin-converting enzyme inhibitors or angiotensin receptor blockers, lipid-lowering drugs, and nonsteroidal anti-inflammatory drugs). Shading indicates 95% CIs.

Lithium therapy initiation, compared with valproate therapy initiation, was not associated with a significantly higher risk of CKD progression (adjusted HR, 1.11; 95% CI, 0.86-1.45) (Figure 1A). In weighted analysis, CKD progression occurred in 8.4% (95% CI, 7.0%-10.0%) of patients initiating lithium therapy and 8.2% (95% CI, 6.8%-9.8%) of patients initiating valproate therapy during 10 years of observation (weighted 10-year absolute risk difference, 0.2%; 95% CI, −2.0% to 2.4.%), which did not differ from zero throughout the follow-up period (Figure 1B).

We identified 770 AKI events (Table 2). There was no difference between groups in risk (weighted HR, 0.88; 95% CI, 0.70-1.10) (Figure 1C). However, the 10-year absolute risk difference was −3.2% (95% CI, −5.6 to −1.1), with a lower risk among patients initiating lithium therapy than those initiating valproate therapy (Figure 1D).

We identified 438 (4.5% of total) new albuminuria events (Table 2). There was no difference between groups in albuminuria risk (weighted HR, 0.99; 95% CI, 0.74-1.33) (Figure 1E). The 10-year absolute risk difference was −1.1% (95% CI, −2.7 to 2.1) (Figure 1F).

The annual rate eGFR decrease was −1.1 mL/min/1.73 m^2^ (95% CI: −1.2 to −1.0 mL/min/1.73 m^2^) for valproate users and −0.9 mL/min/1.73 m^2^ (95% CI, −1.0 to −0.8 mL/min/1.73 m^2^) for lithium users. There were no differences between the rate of eGFR decrease between therapies, with a nonstatistically significant annual mean difference of 0.2 mL/min/1.73 m^2^ slower for lithium users compared with valproate users. Absence of statistically significant or clinically meaningful differences in eGFR slopes was observed across age and baseline eGFR strata (eTable 4 in Supplement 1).

Using overlap weights as an alternative propensity weighting method yielded results similar to our main analysis: lithium vs valproate therapy initiation was associated with an adjusted HR of 1.18 (95% CI, 0.92-1.50) for CKD progression, 0.96 (95% CI, 0.79-1.16) for AKI, and 0.87 (95% CI, 0.69-1.10) for new albuminuria (eTables 5 and 6 in Supplement 1). The frequency of creatinine testing during follow-up was similar in both treatment groups (eTable 7 in Supplement 1).

### Cumulative Use of Lithium vs Valproate

**Figure 2.**
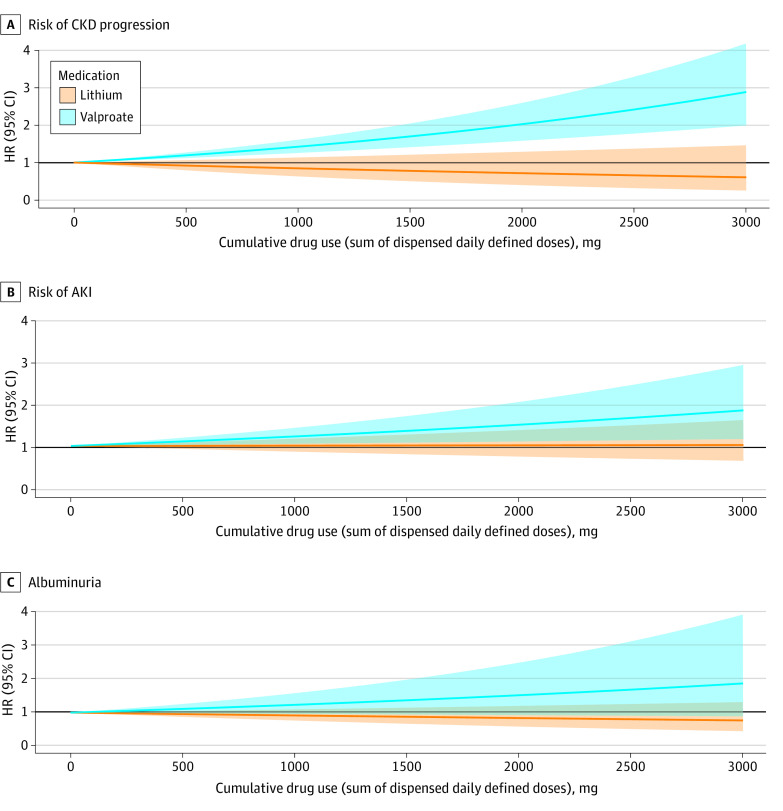
Hazard Ratios (95% CIs) for the Risk of Chronic Kidney Disease (CKD) Progression, Acute Kidney Injury (AKI), or Albuminuria Associated With the Cumulative Use of Lithium and Valproate The x-axis is truncated at 3000 defined daily dosages (DDDs) because this is the maximum DDD in the valproate group. Cumulative dose was calculated as the time updated sum of all dispensed DDDs since therapy initiation and modeled as a time-dependent covariate. Models adjusted for the following variables: age, sex, attained education, baseline estimated glomerular filtration rate, number of hospitalizations during previous year and psychiatry related, number of outpatient contacts during previous year and psychiatry related, number of outpatient contacts during previous year, pregnancy in the 2 years prior, total number of medications in the previous year, comorbidities (bipolar disorder, depression, manic episode, anxiety disorder, mental disorders attributable to psychoactive substance use, schizophrenia spectrum disorders, hypertension, diabetes, acute coronary syndrome, other ischemic heart disease, heart failure, stroke, other cerebrovascular diseases, valve disorders, atrial fibrillation, other arrhythmia, hyperthyroidism, cancer, and liver disease), and ongoing medications (lamotrigine, carbamazepine, first- and second-generation antipsychotic drugs, other mood stabilizers, antidepressants, attention-deficit/hyperactivity disorder medication, drugs used in addictive disorders, opioids and pain medications, antiepileptic drugs, β-blockers, calcium channel blockers, diuretics, angiotensin-converting enzyme inhibitors or angiotensin receptor blockers, lipid-lowering drugs, and nonsteroidal anti-inflammatory drugs). Shaded areas indicate 95% CIs. HR indicates hazard ratio.

Patients who initiated lithium therapy had longer treatment durations (eFigure 4 in Supplement 1) than those who initiated valproate therapy. A total of 15% of patients in the lithium group and 12% in the valproate group had at least one dispensation of the other drug during follow up. Although increasing cumulative lithium use was associated with a graded higher risk of CKD progression (Figure 2A), no association was observed for the cumulative use of valproate. Compared with valproate treatment, every 500 DDDs of lithium dispensed was associated with a 30% higher risk of CKD progression (ratio of HRs, 1.30; 95% CI, 1.09-1.50). No difference was observed between therapies for the risk of AKI (ratio of HRs, 0.91; 95% CI, 0.82-0.99) (Figure 2B) or new albuminuria (ratio of HRs, 0.86; 95% CI, 0.75-0.98) (Figure 2C).

### Association Between Long-Term High vs Low Serum Lithium Levels and Kidney Outcomes

For the 3518 adults who continued to take lithium for at least 1 year (eFigure 5 and eTable 8 in Supplement 1), the median lithium level during the first year of therapy was 0.5 mmol/L (IQR, 0.40-0.53 mmol/L). During therapy, there were 35 443 measurements of lithium recorded, with a median of 8 (IQR, 3-18) measurements per person; most measurements (88%) were less than or equal to 0.8 mmol/L. In total, 11% of lithium measurements (in 30% of individuals) were greater than 0.8 mmol/L, and 3% (in 13% of individuals) were greater than 1.0 mmol/L.

Compared with lower lithium levels, higher levels were associated with tendencies to increasing risk of CKD, with a dose-response relation across lithium thresholds, seen in both baseline (HR, 2.86; 95% CI, 0.97-8.45) and time-varying (HR, 1.77; 95% CI, 0.50-6.31) models (Table 3). The association with risk of AKI was stronger and statistically significant in time-varying models: compared with low lithium levels, the risk of AKI was 2.5-fold higher for a median lithium level greater than 0.8 mmol/L (HR, 2.56; 95% CI, 1.67-3.92) and 3.5-fold higher for a median lithium level greater than 1.0 mmol/L (HR, 3.51; 95% CI, 1.41-8.76) (Table 3). There was no association between higher levels and risk of new albuminuria in either model (Table 3).

**Table 3.** Number of Events and Adjusted HRs for the Association Between Long-Term Serum Lithium and Kidney Outcomes Among People Using Lithium for More Than 1 Year^a^

	No. of events	HR (95% CI)			
		Continuous: per lithium 0.1 mmol/L higher	Lithium >0.8 mmol/L	Lithium >0.9 mmol/L	Lithium >1.0 mmol/L
**CKD progression (n = 3518 participants)**					
Baseline lithium	135	1.06 (0.96-1.17)	1.03 (0.36-2.91)	2.17 (0.87-5.38)	2.86 (0.97-8.45)
Time-varying lithium		1.02 (0.93-1.13)	1.06 (0.50-2.22)	1.24 (0.41-3.76)	1.77 (0.50-6.31)
**Acute kidney injury (n = 3518 participants)**					
Baseline lithium	144	0.98 (0.87-1.10)	0.93 (0.30-2.85)	2.30 (0.80-6.59)	2.47 (0.76-7.99)
Time-varying lithium		1.22 (1.13-1.31)	2.56 (1.67-3.92)	4.33 (2.34-8.00)	3.51 (1.41-8.76)
**Albuminuria (n = 3097 participants)**					
Baseline lithium	101	0.91 (0.81-1.02)	Converge to infinite	Converge to infinite	Converge to infinite
Time-varying lithium		0.91 (0.82-1.02)	0.7 (0.24-2.08)	0.7 (0.16-3.77)	1.4 (0.25-7.94)

Abbreviations: CKD, chronic kidney disease; HR, hazard ratio.

^a^ Output from multivariable Cox proportional hazards regression models were adjusted for the following variables: age, sex, attained education, baseline estimated glomerular filtration rate, number of hospitalizations during previous year and psychiatry related, number of outpatient contacts during previous year and psychiatry related, number of outpatient contacts during previous year, pregnancy in the 2 years prior, total number of medications in the previous year, comorbidities (bipolar disorder, depression, manic episode, anxiety disorder, mental disorders attributable to psychoactive substance use, schizophrenia spectrum disorders, hypertension, diabetes, acute coronary syndrome, other ischemic heart disease, heart failure, stroke, other cerebrovascular diseases, valve disorders, atrial fibrillation, other arrhythmia, hyperthyroidism, cancer, and liver disease), and ongoing medications (lamotrigine, carbamazepine, first- and second-generation antipsychotic drugs, other mood stabilizers, antidepressants, attention-deficit/hyperactivity disorder medication, drugs used in addictive disorders, opioids and pain medications, antiepileptic drugs, β-blockers, calcium channel blockers, diuretics, angiotensin-converting enzyme inhibitors or angiotensin receptor blockers, lipid-lowering drugs, and nonsteroidal anti-inflammatory drugs).

## Discussion

We found no difference in the risk of CKD progression for new use of lithium compared with valproate and that a higher cumulative dose of lithium, compared with valproate, was modestly associated with the risk of CKD progression. The absolute risks were low, and there was no difference between therapies within a 10-year horizon. Rate of change of GFR, a post hoc outcome, was also not different between groups, with a modest annual mean difference of 0.2 mL/min/1.73 m^2^. New users of lithium were no more likely to develop albuminuria. Toxic lithium levels (>1.0 mmol/L) were uncommon. However, people with higher lithium levels were at higher risk of CKD and AKI.

Because it has been suggested^22^ that modern lithium treatment (recommended levels of 0.6-0.8 mmol/L; up to 1.0 mmol/L if insufficient response and good tolerance^23^) has minimized the risk of lithium-induced KRT since the 1960 to 1980s, we compared our results with studies after the 2012 meta-analysis.^7^ In our study of more than 35 000 lithium levels, 3% were greater than 1.0 mmol/L, a very low proportion, and similar to a UK report.^5^

Two studies^9,13^ comparing use vs nonuse of lithium reached opposing results. Shine et al^9^ identified 2795 patients undergoing lithium testing at a UK National Health Service trust (1985-2014). Compared with random health care users, those tested for lithium had a higher risk (HR, 1.93; 95% CI, 1.76-2.12) of having 1 detected annual GFR measurement less than 60 mL/min/1.73 m^2^ during follow-up. Kessing et al^13^ studied 10 591 people with bipolar disorder or manic episode in a Danish population-based register (2000-2011). Use of lithium, compared with no use, and a high number of lithium dispensations (≥60) were associated with the risk of receiving a CKD diagnosis (HR, 2.54; 95% CI, 1.81-3.57). Because the risk of starting KRT (HR, 0.32; 95% CI, 0.09-1.11) did not differ, the authors interpreted that increased surveillance among lithium users led to higher disease recognition (ie, a false-positive finding).

Two studies^5,10^ with an active comparator also reached somewhat opposing results. Clos et al^5^ evaluated 305 new users of lithium and 815 new users of another first-line drug for the treatment of affective disorders, finding that the annual decrease in eGFR during median 6-year follow-up did not differ significantly between the lithium group (1.3 mL/min/1.73 m^2^) and the comparator group (0.9 mL/min/1.73 m^2^). However, lithium levels greater than 0.8 mmol/L were associated with a decrease in eGFR. In our study, rates of eGFR decrease were of comparable magnitude and were not significantly different between groups. Rej et al^10^ evaluated 3113 lithium users older than 65 years propensity score matched 1:1 to 3113 valproate users from Ontario, Canada (2007-2015). Lithium use was associated with a modestly increased risk of a 30% decrease in eGFR (HR, 1.14; 95% CI, CI 1.02-1.27), but absolute risks were low and similar (6.4 events per 100 person-years in the lithium group and 5.8 events per 100 person-years in the valproate group).

### Strengths and Limitations

Our work is consistent with these aforementioned findings of low absolute risks and has some additional strengths: a focus on new users of 2 medications with similar indications and use of inverse probability of treatment weights to mitigate confounding; demonstration that both groups have similar rates of GFR testing over time, reducing the possibility of detection bias; use of a robust method to assess GFR decrease^24^; the largest sample size to date evaluating the impact of lithium levels; the inclusion of albuminuria as a novel study outcome; and our use of rolling 1-year assessments to evaluate long-term toxic effects. Our large sample size, inclusion of all ages, and duration of follow-up improve the power and generalizability of our study.

The exploration of AKI risk in our study is novel. We attribute the reduced risk of AKI for lithium vs valproate in the intention-to-treat analysis to chance because the differences became more marked at distal time points when most patients were no longer taking the drug and because there is no association in the analysis of cumulative dose. The finding of a strong association between lithium levels and AKI is more credible and extends knowledge from a previous report.^12,25^ In a 2018 evaluation^25^ of almost 200 000 reports of possible drug-induced AKI from the US Food and Drug Administration Adverse Event Reporting System, 675 events were attributed to lithium, with a reporting odds ratio, a measure of reporting disproportionality, of 8.86 (95% CI, 8.15-9.64). Kirkham et al^12^ selected 699 patients from the Norfolk database (2002-2013) to evaluate a possible association between short-term exposure to toxic lithium levels and rapid GFR decrease. They found that a single lithium level greater than 1 mmol/L was associated with a 3-month GFR decrease, compared with patients with lithium levels of 0.8 mmol/L or less. It is possible that repeat AKI episodes explain the subsequent CKD progression risk, perhaps in keeping with the histologic features of chronic tubulointerstitial nephropathy with focal segmental glomerulosclerosis.^4^ Because AKI events may be unnoticed or not result in clinical diagnosis,^26^ the evaluation of creatinine elevations according to current AKI classification systems is a strength of our study.^27^

We also recognize a number of limitations to our study. Our study included people in Stockholm, and extrapolation to other health systems or to more ethnically diverse societies should be made cautiously. Valproate may not be a perfect comparator because it has broader indications than lithium, and we identified case reports of valproate-induced Fanconi syndrome.^28^ Lithium measurements were not assessed at regular time points but by indication, and whether they were trough levels is not known.^29^ We selected lithium toxicity thresholds a priori. In light of the results, examining lower thresholds and the exposure time above threshold calculated from interpolated data would be important in future work. We controlled for available covariates but cannot completely eliminate bias because of unmeasured or unknown confounding. Approximately 15% of patients in each group had at least 1 dispensation of the other drug; although this is a relatively low crossover, the effect of any crossover is to bias results toward the null. Our study cannot distinguish among different causal mechanisms; our findings could reflect low-level toxic effects manifesting in some patients or idiosyncratic rare events, such as the development of interstitial nephritis or glomerular disease.^4,29^ In addition, the median duration of follow-up in our cohort is short relative to the natural history of lithium nephropathy and short compared with a life lived with bipolar disorder. Our results should be considered valid within a 10-year horizon, reflecting clinically prevalent patterns of lithium use during the period 2007 to 2018.

## Conclusions

This cohort study provides, to our knowledge, the best estimates to date of the possible long-term effects of lithium therapy, supporting the hypothesis that longer duration may be a risk factor for CKD and identifying for the first time that high levels are a risk factor for AKI. However, we also found that absolute risks were low and propose that these risks need to be offset by considering the effectiveness and antisuicidal benefits of lithium.^2^ These results inform starting, monitoring, and adjusting lithium therapy as recommended by guidelines and to avoid lithium toxicity. Further work is needed to understand how to manage those few patients who experience progression or new-onset CKD.

## Notes

Supplement 1. **eMethods.** Definition of Study Outcomes and Statistical Analysis **eTable 1.** Definition of Study Covariates, Comorbidities **eTable 2.** Definition of Study Covariates, Ongoing Medications **eTable 3.** Baseline Characteristics of Additional Variables (Beyond Those Reported in Table 1) Used in the Propensity Score Calculation of Patients Initiating Lithium or Valproate Treatment Between 2007 and 2018 in Stockholm **eTable 4.** Annual eGFR Decline and Mean Difference in eGFR Decline by Treatment Assignment, Overall and by Subgroups **eTable 5.** Baseline Characteristics of Patients Initiating Lithium or Valproate Treatment Between 2007 and 2018 in Stockholm Before and After Overlap Weighting (OW) **eTable 6.** Number of Events, Incidence Rates, Adjusted Hazard Ratios and 10-Year Absolute Risk Reduction for the Association Between Valproate vs Lithium and Kidney Outcomes Applying Overlapping Weights **eTable 7.** Sensitivity Analyses: Frequency of Creatinine Measurements During Follow-up, Overall and Within Yearly Intervals **eTable 8.** Baseline Characteristics of Patients Who Initiated Lithium Therapy Between 2007 and 2018 in Stockholm and Stayed on Therapy for at Least One Year, Overall and by Serum Level Categories **eFigure 1.** Study Design Diagram **eFigure 2.** Flowchart of Study Inclusion **eFigure 3.** Pattern of Lithium and Valproate Prescriptions Over Time **eFigure 4.** Distribution of the Time Elapsed Between First and Last Lithium Dispensation (Panel A) and Valproate (Panel B) **eFigure 5.** Distribution of Routine Lithium Measurements Among 3518 Individuals Who Stayed on Lithium Therapy for at Least One Year
eReferences

Supplement 2.
Data Sharing Statement
