# Supplementary material for: Absolute and Relative Risks of Kidney Outcomes Associated With Lithium vs Valproate Use in Sweden
Source: JAMA Netw Open. 2023 Jul 7;6(7):e2322056. doi: 10.1001/jamanetworkopen.2023.22056 (PMC10329212; doi:10.1001/jamanetworkopen.2023.22056)
Supplement: Supplement 1. — eMethods. Definition of Study Outcomes and Statistical Analysis eTable 1. Definition of Study Covariates, Comorbidities eTable 2. Definition of Study Covariates, Ongoing Medications eTable 3. Baseline Characteristics of Additional Variables (Beyond Those Reported in Table 1) Used in the Propensity Score Calculation of Patients Initiating Lithium or Valproate Treatment Between 2007 and 2018 in Stockholm eTable 4. Annual eGFR Decline and Mean Difference in eGFR Decline by Treatment Assignment, Overall and by Subgroups eTable 5. Baseline Characteristics of Patients Initiating Lithium or Valproate Treatment Between 2007 and 2018 in Stockholm Before and After Overlap Weighting (OW) eTable 6. Number of Events, Incidence Rates, Adjusted Hazard Ratios and 10-Year Absolute Risk Reduction for the Association Between Valproate vs Lithium and Kidney Outcomes Applying Overlapping Weights eTable 7. Sensitivity Analyses: Frequency of Creatinine Measurements During Follow-up, Overall and Within Yearly Intervals eTable 8. Baseline Characteristics of Patients Who Initiated Lithium Therapy Between 2007 and 2018 in Stockholm and Stayed on Therapy for at Least One Year, Overall and by Serum Level Categories eFigure 1. Study Design Diagram eFigure 2. Flowchart of Study Inclusion eFigure 3. Pattern of Lithium and Valproate Prescriptions Over Time eFigure 4. Distribution of the Time Elapsed Between First and Last Lithium Dispensation (Panel A) and Valproate (Panel B) eFigure 5. Distribution of Routine Lithium Measurements Among 3518 Individuals Who Stayed on Lithium Therapy for at Least One Year eReferences [file jamanetwopen-e2322056-s001.pdf]

Supplementary Online Content

Bosi A, Clase CM, Ceriani L, et al. Absolute and relative risks of kidney outcomes associated with lithium vs valproate use in Sweden. *JAMA Netw Open*. 2023;6(7):e2322056. doi:10.1001/jamanetworkopen.2023.22056

eMethods. Definition of Study Outcomes and Statistical Analysis
eTable 1. Definition of Study Covariates, Comorbidities
eTable 2. Definition of Study Covariates, Ongoing Medications
eTable 3. Baseline Characteristics of Additional Variables (Beyond Those Reported in Table 1) Used in the Propensity Score Calculation of Patients Initiating Lithium or Valproate Treatment Between 2007 and 2018 in Stockholm
eTable 4. Annual eGFR Decline and Mean Difference in eGFR Decline by Treatment Assignment, Overall and by Subgroups
eTable 5. Baseline Characteristics of Patients Initiating Lithium or Valproate Treatment Between 2007 and 2018 in Stockholm Before and After Overlap Weighting (OW)
eTable 6. Number of Events, Incidence Rates, Adjusted Hazard Ratios and 10-Year Absolute Risk Reduction for the Association Between Valproate vs Lithium and Kidney Outcomes Applying Overlapping Weights
eTable 7. Sensitivity Analyses: Frequency of Creatinine Measurements During Follow-up, Overall and Within Yearly Intervals
eTable 8. Baseline Characteristics of Patients Who Initiated Lithium Therapy Between 2007 and 2018 in Stockholm and Stayed on Therapy for at Least One Year, Overall and by Serum Level Categories
eFigure 1. Study Design Diagram
eFigure 2. Flowchart of Study Inclusion
eFigure 3. Pattern of Lithium and Valproate Prescriptions Over Time
eFigure 4. Distribution of the Time Elapsed Between First and Last Lithium Dispensation (Panel A) and Valproate (Panel B)
eFigure 5. Distribution of Routine Lithium Measurements Among 3518 Individuals Who Stayed on Lithium Therapy for at Least One Year
eReferences

This supplementary material has been provided by the authors to give readers additional information about their work.

© 2023 Bosi A et al. *JAMA Network Open.*

eMethods. Definition of Study Outcomes and Statistical Analysis

To reduce outcome misclassification bias owing to intrinsic eGFR variability, and to confirm whether eGFR declines were sustained over time, we used a linear interpolation method [1]. In brief, for each individual we fitted a linear regression line through all outpatient eGFR measurements. Sustained eGFR decline ≥30% from baseline was defined as a negative linear regression slope that crossed the 30% decline threshold before the last measurement: the time at which this occurred was used as the outcome in time-to-event analyses. The date of progression of CKD was defined by this event, or as the date of initiation of maintenance dialysis or kidney transplantation (ascertained by linkage with the Swedish Renal Registry) or date of death attributed to CKD as primary reporting cause (ICD-10 code N18).

AKI was identified by a combination of issued diagnoses (ICD-10 code: N17) in outpatient or hospital care and transient creatinine elevations during hospitalization, using KDIGO criteria [2] (ie, rise in creatinine ≥ 26 µmol/L over 48 hours or >1.5 times baseline within 7 days) or death attributed to AKI as a primary cause (ICD-10 code N17). At each hospitalization, we calculated the baseline eGFR as the mean of all creatinine measurements in the previous year, excluding the 7 days immediately before hospitalization.

For the outcome of albuminuria extracted all performed measurements of dipstick albuminuria /proteinuria and urinary albumin/protein to creatinine ratio. These measurements were then categorized according to KDIGO A-stages using the thresholds defined for each method by our central laboratories. KDIGO A-stages include mildly increased (A1, ACR <30 mg/g), moderately increased (A2, ACR 30-300 mg/g), and severely increased albuminuria (A3, ACR >300 mg/g). We identified the earliest albuminuria test recorded prior to study baseline and excluded for this analysis all patients with albuminuria measurement in the category A2 or A3. We assumed category A1 in those without a history of recorded albuminuria. The study outcome, new albuminuria, was defined by the presence of at least two albuminuria measurements in the categories A2 or A3 during follow up, and the date of the first new albuminuria test was the event date.

We used inverse probability of treatment weighting (IPTW) to control for baseline confounding. Using a multivariable logistic regression model, we estimated the probability of receiving lithium versus valproate as a function of all baseline covariates listed above. Weighting was considered appropriate if the standardized mean difference (SMD) between treatment groups was <0.1. Weights were stabilized to increase precision by adding the marginal probability of treatment to the numerator of the weights. Robust variance estimation was used to calculate confidence intervals after weighting.

© 2023 Bosi A et al. *JAMA Network Open.*

eTable 1. Definition of Study Covariates, Comorbidities

Baseline comorbidities	ICD-code beginning with
Bipolar Disorder	F31
Hyperthyroidism	E05
Other ischemic heart disease	I201, I208, I209, I24, I25
Depression	F32,F33
Manic episode	F30
Anxiety Disorder	F40,F41
Mental disorders due to psychoactive substance use	F10
Schizophrenia spectrum disorders	F20-F29
Hypertension	I10-I15
Diabetes Mellitus	E10,E11,E12,E13,E14
Acute coronary syndrome	I200, I21-I22
Other ischemic heart disease	I201, I208, I209, I24, I25
Heart failure	I110, I130, I132, I50
Valve disorders	I34-I37
Stroke	I60-I64, I693, I698, I694
Other cerebrovascular disease	I65-I69, G45 (excl G454), G46
Atrial fibrillation	I48
Other arrhythmia	I44-I47, I49
Peripheral vascular disease	I70, I72, I73
Pregnancy in the two years prior	Z33
Cancer in previous three years	C00-C43, C45-C97

© 2023 Bosi A et al. *JAMA Network Open.*

eTable 2. Definition of Study Covariates, Ongoing Medications

Medication	ATC code beginning with
Lithium	N05AN01
Valproate	N03AG01
Lamotrigine	N03AX09
Carbamazepine	N03AF01
First generation antipsychotic drugs	N05AA, N05AB, N05AD, N05AF
Second generation antipsychotic drugs	N05AE, N05AH, N05AX
Other mood stabilizers	N05AX08, N05AX12, N05AX13, N05AX15, N05AX16
Antidepressants	N06A
Attention-deficit/hyperactivity disorder medication	N06B
Drugs used in addictive disorders	N07B
Opioids and pain medications	N02A
Antiepileptic drugs	N03A (excluding N03AG01, N03AX09 and N03AF01)
Beta blockers	C07
Calcium-channel blockers	C08
Diuretic	C03
ACEi/ARB	C09A, C09B, C09C, C09D
Lipid-lowering drug	C10
NSAID	M01A

ACEi angiotensin converting enzyme inhibitors; ARBs angiotensin-receptor blockers; NSAID non-steroidal anti-inflammatory drug;

© 2023 Bosi A et al. *JAMA Network Open.*

eTable 3. Baseline Characteristics of Additional Variables (Beyond Those Reported in Table 1) Used in the Propensity Score Calculation of Patients Initiating Lithium or Valproate Treatment Between 2007 and 2018 in Stockholm

		Before IPTW			After IPTW		
	**Overall**	**Valproate**	**Lithium**	**SMD**	**Valproate**	**Lithium**	**SMD**
**Pregnancy in the two years prior**	6 (0)	4 (0)	2 (0)	0.014	3 (0)	2 (0)	0.006
**Number of hospitalizations during previous year**				0.291			0.072
**0**	4597 (42)	2040 (36)	2557 (48)		2497 (42)	2141 (44)	
**1**	2344 (21)	1233 (22)	1111 (21)		1258 (21)	1033 (21)	
**2**	1525 (14)	805 (14)	720 (14)		855 (14)	655 (14)	
**3**	884 (8)	544 (10)	340 (6)		460 (8)	422 (9)	
**>3**	1596 (15)	1016 (18)	580 (11)		851 (14)	605 (13)	
**Number of hospitalizations during previous year (Psychiatry related)**				0.199			0.038
**0**	6374 (58)	3540 (63)	2834 (53)		3395 (57)	2701 (56)	
**1**	2003 (18)	905 (16)	1098 (21)		1094 (19)	935 (19)	
**2**	1123 (10)	487 (9)	636 (12)		660 (11)	545 (11)	
**3**	594 (5)	288 (5)	306 (6)		305 (5)	271 (6)	
**>3**	852 (8)	418 (7)	434 (8)		466 (8)	404 (8)	
**Number of outpatient contacts during previous year**				0.366			0.070
**0-3**	2845 (26)	1828 (32)	1017 (19)		1463 (25)	1078 (22)	
**3-8**	2656 (24)	1462 (26)	1194 (23)		1383 (23)	1104 (23)	
**8-17**	2549 (23)	1124 (20)	1425 (27)		1435 (24)	1276 (26)	
**>17**	2896 (27)	1224 (22)	1672 (32)		1640 (28)	1397 (29)	
**Number of outpatient contacts during previous year (Psychiatry related)**				0.656			0.104
**0-3**	5199 (48)	3527 (63)	1672 (32)		2662 (45)	1951 (40)	
**3-8**	1945 (18)	755 (13)	1190 (22)		1068 (18)	1022 (21)	
**8-17**	1754 (16)	625 (11)	1129 (21)		1014 (17)	868 (18)	
**>17**	2048 (19)	731 (13)	1317 (25)		1176 (20)	1016 (21)	
**Beta blockers**	1748 (16)	1166 (21)	582 (11)	0.269	925 (16)	732 (15)	0.015
**Calcium-channel blockers**	804 (7)	544 (10)	260 (5)	0.184	428 (7)	293 (6)	0.048
**Diuretics**	1015 (9)	779 (14)	236 (4)	0.330	524 (9)	308 (6)	0.095
**ACEi/ARB**	1489 (14)	1030 (18)	459 (9)	0.285	769 (13)	569 (12)	0.038
**Lipid lowering drug**	1279 (12)	914 (16)	365 (7)	0.295	675 (11)	474 (10)	0.053
**NSAID**	2376 (22)	1278 (23)	1098 (22)	0.048	1248 (21)	1143 (24)	0.059
**Antiepileptic drugs**	1853 (17)	1182 (21)	671 (13)	0.224	937 (16)	726 (15)	0.024
**Total Medications**				0.214			0.032
**0-5**	8200 (75)	3973 (71)	4227 (80)		4445 (75)	3578 (74)	
**6-10**	2546 (23)	1537 (27)	1009 (19)		1361 (23)	1181 (24)	
**>11**	193 (2)	128 (2)	72 (1)		114 (2)	97 (2)	

© 2023 Bosi A et al. *JAMA Network Open.*

eTable 4. Annual eGFR Decline and Mean Difference in eGFR Decline by Treatment Assignment, Overall and by Subgroups

	Rate of eGFR decline, in mL/min/1.73m^2/year		Mean difference in rate of eGFR decline, in mL/min/1.73m^2/year Lithium vs Valproate users (95% CI)
	**Valproate new users (95% CI)**	**Lithium new users (95% CI)**	
Overall	-1.1 (-1.2 - -1.0)	-0.9 (-1.0 - -0.8)	0.2 (0.0 - 0.3)
Age strata			
≥ 65 years	-0.8 (-1.1 - -0.6)	-1.1 (-1.5 - -0.7)	-0.3 (-0.7 - 0.2)
<65 years	-1.1 (-1.2 - -1.0)	-0.9 (-1.0 - -0.8)	0.2 (0.1 - 0.3)
eGFR strata			
≥ 60 ml/min/1.73 m^2/year	-1.0 (-1.1 - -0.9)	-0.8 (-0.9 - -0.7)	0.2 (0.1 - 0.3)
<60 ml/min^2/1.73 m^2/year	-0.9 (-1.5 - -0.3)	-1.0 (-1.9 – 0.0)	-0.1 (- 1.2 - 1.0)

Legend: Negative values denote loss of eGFR and positive values gain of eGFR over time

© 2023 Bosi A et al. *JAMA Network Open.*

eTable 5. Baseline Characteristics of Patients Initiating Lithium or Valproate Treatment Between 2007 and 2018 in Stockholm Before and After Overlap Weighting (OW)

		Before OW			After OW		
	Overall	Valproate	Lihtium	SMD	Valproate	Lithium	SMD
**Number of individuals**	10946	5638	5308		1869	1869	<0.001
**Age, median (IQR)**	45 [32, 59]	49 [34, 66]	41 [30, 53]	0.464	43 [30, 55]	43 [31, 55]	<0.001
**Women**	6227 (57)	2909 (52)	3318 (62.5)	0.222	1067 (57)	1067 (57)	<0.001
**Men**	4719 (43)	2729 (48)	1990 (38)		801 (43)	801 (43)	
**eGFR, median (IQR), ml/min/1.73m^2/year**	99 [85, 112]	97 [82, 111]	101 [88, 113]	0.213	101 [88, 113]	101 [87, 112]	<0.001
**eGFR category**				0.287			0.074
**eGFR<60 ml/min/1.73m^2/year**	503 (5)	413 (7)	90 (2)		62 (3)	39 (2)	
**eGFR>60 ml/min/1.73m^2/year**	10443 (95)	5225 (93)	5218 (98)		1806 (97)	1829 (98)	
**Bipolar Disorder**	4053 (37)	1034 (18)	3019 (57)	0.867	720 (39)	720 (39)	<0.001
**Depression**	6115 (56)	2293 (41)	3822 (72)	0.666	1156 (62)	1156 (62)	<0.001
**Manic episode**	560 (5)	207 (4)	353 (7)	0.135	115 (6)	115 (6)	<0.001
**Anxiety Disorder**	4638 (42)	1882 (33)	2756 (52)	0.382	892 (48)	892 (48)	<0.001
**Mental disorders due to psychoactive substance use**	2022 (18)	1000 (18)	1022 (19.3)	0.039	388 (21)	388 (21)	<0.001
**Schizophrenia spectrum disorders**	1869 (17)	982 (17)	887 (17)	0.019	381 (20)	381 (20)	<0.001
**Hyperthyroidism**	131 (1)	76 (1)	55 (1)	0.029	20 (1)	20 (1)	<0.001
**Hypertension**	2217 (20)	1515 (27)	702 (13)	0.346	299 (16)	299 (16)	<0.001
**Diabetes Mellitus**	858 (8)	593 (11)	265 (5)	0.208	122 (7)	122 (7)	<0.001
**Acute coronary syndrome**	308 (3)	261 (5)	47 (1)	0.230	26 (1)	26 (1)	<0.001
**Other ischemic heart disease**	561 (5)	458 (8)	103 (2)	0.286	52 (3)	52 (3)	<0.001
**Heart failure**	406 (4)	359 (6)	47 (1)	0.296	30 (2)	30 (2)	<0.001
**Stroke**	960 (9)	872 (16)	88 (2)	0.509	54 (3)	54 (3)	<0.001
**Other cerebrovascular disease**	887 (8)	784 (14)	103 (2)	0.454	57 (3)	57 (3)	<0.001
**Atrial fibrillation**	520 (5)	444 (8)	76 (1)	0.310	38 (2)	38 (2)	<0.001
**Arrhythmia**	491 (5)	342 (6)	149 (3)	0.159	67 (4)	67 (4)	<0.001
**Peripheral vascular disease**	218 (2)	168 (3)	50 (1)	0.147	23 (1)	23 (1)	<0.001
**Valve disorders**	109 (1)	91 (2)	18 (0)		10 (1)	10 (1)	<0.001
**Liver disease**	395 (4)	231 (4)	164 (3)	0.054	68 (4)	68 (4)	<0.001
**Cancer**	535 (5)	439 (8)	96 (2)	0.282	46 (3)	46 (3)	<0.001
**Pregnancy in the two years prior**	6 (0.1)	4 (0.1)	2 (0.0)	0.014	1 (0.0)	1 (0.0)	<0.001
**Number of hospitalizations during previous year**				0.291			<0.001
**0**	4597 (42)	2040 (36)	2557 (48)		854 (46)	854 (46)	
**1**	2344 (21)	1233 (22)	1111 (21)		395 (21)	395 (21)	
**2**	1525 (14)	805 (14)	720 (14)		249 (13)	249 (13)	
**3**	884 (8)	544 (10)	340 (6)		137 (7)	137 (7)	
**>3**	1596 (15)	1016 (18)	580 (11)		234 (13)	234 (13)	
**Number of hospitalizations during previous year(Psychiatry related)**				0.199			<0.001
**0**	6374 (58)	3540 (63)	2834 (53)		1007 (54)	1007 (54)	

© 2023 Bosi A et al. *JAMA Network Open.*

**1**	2003 (18)	905 (16)	1098 (21)		367 (20)	367 (20)	
**2**	1123 (10)	487 (9)	636 (12)		218 (12)	218 (12)	
**3**	594 (5)	288 (5)	306 (6)		112 (6)	112 (6)	
**>3**	852 (8)	418 (7)	434 (8)		164 (9)	164 (9)	
**Number of outpatient contacts during previous year**				0.366			<0.001
**0-3**	2845 (26)	1828 (32)	1017 (19)		459 (25)	459 (25)	
**3-8**	2656 (24)	1462 (26)	1194 (23)		437 (23)	437 (23)	
**8-17**	2549 (23)	1124 (20)	1425 (27)		448 (24)	448 (24)	
**>17**	2896 (27)	1224 (22)	1672 (32)		525 (28)	525 (28)	
**Number of outpatient contacts during previous year(Psychiatry related)**				0.656			<0.001
**0-3**	5199 (48)	3527 (63)	1672 (32)		776 (42)	776 (42)	
**3-8**	1945 (18)	755 (13)	1190 (22)		367 (20)	367 (20)	
**8-17**	1754 (16)	625 (11)	1129 (21)		335 (18)	335 (18)	
**>17**	2048 (19)	731 (13)	1317 (25)		390 (21)	390 (21)	
**Lamotrigine**	2375 (22)	789 (14)	1586 (30)	0.391	399 (21)	399 (21)	<0.001
**Carbamazipine**	605 (6)	542 (10)	63 (1)	0.379	41 (2)	41 (2)	<0.001
**First generation antipsychotic drugs**	1727 (16)	921 (16)	806 (15)	0.032	348 (19)	348 (19)	<0.001
**Second generation antipsychotic drugs**	4891 (45)	1877 (33)	3014 (57)	0.486	915 (49)	915 (49)	<0.001
**Other mood stabilizers**	1468 (13)	647 (12)	821 (16)	0.117	275 (15)	275 (15)	<0.001
**Antidepressants**	6757 (62)	2850 (51)	3907 (74)	0.489	1244 (67)	1244 (67)	<0.001
**Attention-deficit/hyperactivity disorder medication**	793 (7)	369 (7)	424 (8)	0.056	156 (8)	156 (8)	<0.001
**Drugs used in addictive disorders**	696 (6)	356 (6)	340 (6)	0.004	140 (8)	140 (8)	<0.001
**Opioids and pain medications**	2325 (21)	1362 (24)	963 (18)	0.148	385 (21)	385 (21)	<0.001
**Beta blockers**	1748 (16)	1166 (21)	582 (11)	0.269	239 (13)	239 (13)	<0.001
**Calcium-channel blockers**	804 (7)	544 (10)	260 (5)	0.184	111 (6)	111 (6)	<0.001
**Diuretics**	1015 (9)	779 (14)	236 (4)	0.330	110 (6)	110 (6)	<0.001
**ACEi/ARB**	1489 (14)	1030 (18)	459 (9)	0.285	199 (11)	199 (11)	<0.001
**Lipid lowering drug**	1279 (12)	914 (16)	365 (7)	0.295	164 (9)	164 (9)	<0.001
**NSAID**	2376 (22)	1278 (23)	1098 (21)	0.048	420 (23)	420 (23)	<0.001
**Antiepileptic drugs**	1853 (17)	1182 (21)	671 (13)	0.224	280 (15)	280 (15)	<0.001
**Total Medications**				0.214			<0.001
**0-5**	8200 (74)	3973 (71)	4227 (80)		1396 (75)	1396 (75)	
**6-10**	2546 (23)	1537 (27)	1009 (19)		435 (23)	435 (23)	
**>11**	200 (2)	128 (2)	72 (1)		38 (2)	38 (2)	
**Education**				0.356			<0.001
**Compulsory school**	2137 (20)	1397 (25)	740 (14)		344 (18)	344 (18)	
**Secondary school**	4463 (41)	2302 (41)	2161 (41)		791 (42)	791 (42)	
**University**	4142 (38)	1783 (32)	2359 (44)		708 (38)	708 (38)	
**Missing**	204 (2)	156 (3)	48 (0.9)		26 (1)	26 (1)	

© 2023 Bosi A et al. *JAMA Network Open.*

eTable 6. Number of Events, Incidence Rates, Adjusted Hazard Ratios and 10-Year Absolute Risk Reduction for the Association Between Valproate vs Lithium and Kidney Outcomes Applying Overlapping Weights

	No of Events (IR/ 1000 person-years)*	Follow-up, years Median (IQR)	10-y Absolute Risk (95% CI)	Adj HR (95% CI)
CKD progression				
Lithium	182 (6.93)	4.3 (1.9-7.8)	8.4 (6.9-9.9)	1.18 (0.92-1.50)
Valproate	247 (8.95)	4.4 (1.7-7.8)	7.7 (6.2-9.3)	REF
Acute kidney injury				
Lithium	234 (9.01)	4.2 (1.8-7.8)	10 (8.6-11.4)	0.96 (0.79-1.16)
Valproate	536 (20.1)	4.2 (1.6-7.7)	10.4 (8.6-12)	REF
Albuminuria				
Lithium	166 (6.91)	4.4 (2.0-8.0)	6.6 (5.6-7.9)	0.87 (0.69-1.10)
Valproate	272 (11.01)	4.6 (1.8-8.0)	7.8 (6.5-9.0)	REF

* Number of events, incidence rates were calculated in the original, unweighted population; Adj, adjusted;CKD, chronic kidney disease; AKI, acute kidney injury Analyses were weighted for the following variables: age, sex, attained education, baseline eGFR, number of hospitalizations during previous year and psychiatry related, number of outpatient contacts during previous year and psychiatry related, number of outpatient contacts during previous year, total number of medications in the previous year, pregnancy in the two years prior, comorbidities (bipolar disorder, depression, manic episode, anxiety disorder, mental disorders due to psychoactive substance use, schizophrenia spectrum disorders, hypertension, diabetes mellitus, acute coronary syndrome, other ischemic heart disease, heart failure, stroke, other cerebrovascular diseases, valve disorders, atrial fibrillation, other arrhythmia, hyperthyroidism, cancer, liver disease) and ongoing medications (lamotrigine, carbamazipine, first and second generation antipsychotic drugs, other mood stabilizers, antidepressants, attention-deficit/hyperactivity disorder medication, drugs used in addictive disorders, opioids and pain medications, antiepileptic drugs, beta blockers, calcium-channel blockers, diuretic, ACEi/ARB, lipid lowering drugs and NSAID).

© 2023 Bosi A et al. *JAMA Network Open.*

eTable 7. Sensitivity Analyses: Frequency of Creatinine Measurements During Follow-up, Overall and Within Yearly Intervals

treatment	Number of patients	Follow up median [IQR] (year)	Total number of creatinine measurements	Patient years	Crude incidence proportion (creatinine measurements per patient year)
**New users of Lithium or Valproate**					
**Overall**					
Valproate	5638	4.6 (1.8,8.1)	76234	28229	2.70 (2.68,2.72)
Lithium	5308	4.4 (2.0,8.0)	73922	26691	2.77 (2.75,2.79)
**Years 1-3**					
Valproate	5638	3.0 (1.8,3.0)	39799	13404	2.97 (2.94,3.00)
Lithium	5308	3.0 (2.0,3.0)	40069	12819	3.13 (3.10,3.16)
**Years 3-6**					
Valproate	3589	3.0 (1.8,3.0)	20201	8521	2.37 (2.34,2.40)
Lithium	3332	3.0 (1.7,3.0)	18924	7852	2.41 (2.38,2.44)
**Years 6-9**					
Valproate	2130	2.9 (1.5,3.0)	11579	4793	2.42 (2.37,2.46)
Lithium	1979	3.0 (1.5,3.0)	11213	4466	2.51 (2.46,2.56)
**Years 9-12**					
Valproate	1031	1.5 (0.8,2.2)	4655	1511	3.08 (2.99,3.17)
Lithium	1030	1.5 (0.8,2.3)	3716	1554	2.39 (2.31,2.47)

© 2023 Bosi A et al. *JAMA Network Open.*

eTable 8. Baseline Characteristics of Patients Who Initiated Lithium Therapy Between 2007 and 2018 in Stockholm and Stayed on Therapy for at Least One Year, Overall and by Serum Level Categories

	Overall	s-Li <= 0.8 mmol/L	s-Li >0.8 mmol/L	SMD	s-Li <= 1.0 mmol/L	s-Li >1.0 mmol/L	SMD
**Number of individuals**	3518	3465	53		3511	7	
**Age, median (IQR)**	40 [30, 52]	40 [30, 52]	47 [29, 58]	0.271	40 [30, 52]	56 [50, 62]	1.189
**Women**	2211 (62.8)	2183 (63.0)	28 (52.8)	0.207	2205 (62.8)	6 (85.7)	0.543
**eGFR, median (IQR), ml/min/1.73m^2/year**	101 [88, 113]	101 [88, 113]	99 [84, 117]	0.114	101 [88, 113]	85 [68, 105]	0.694
**eGFR category**				0.302			
**eGFR<60 ml/min/1.73m^2/year**	52 (1.5)	48 (1.4)	4 (7.5)		52 (1.5)	0 (0.0)	0.173
**eGFR>60 ml/min/1.73m^2/year**	3466 (98.5)	3417 (98.6)	49 (92.5)		3459 (98.5)	7 (0.0)	
**Depression**	2519 (71.6)	2488 (71.8)	31 (58.5)	0.282	2513 (71.6)	6 (85.7)	0.350
**Manic episode**	266 (7.6)	258 (7.4)	8 (15.1)	0.244	265 (7.5)	1 (14.3)	0.217
**Anxiety Disorder**	1736 (49.3)	1709 (49.3)	27 (50.9)	0.032	1731 (49.3)	5 (71.4)	0.464
**Mental disorders due to psychoactive substance use**	951 (27.0)	935 (27.0)	16 (30.2)	0.071	949 (27.0)	2 (28.6)	0.034
**Schizophrenia spectrum disorders**	624 (17.7)	609 (17.6)	15 (28.3)	0.257	623 (17.7)	1 (14.3)	0.094
**Hyperthyroidism**	36 (1.0)	36 (1.0)	0 (0.0)	0.145	36 (1.0)	0 (0.0)	0.144
**Hypertension**	452 (12.8)	440 (12.7)	12 (22.6)	0.263	450 (12.8)	2 (28.6)	0.396
**Diabetes Mellitus**	177 (5.0)	168 (4.8)	9 (17.0)	0.397	176 (5.0)	1 (14.3)	0.318
**Acute coronary syndrome**	23 (0.7)	23 (0.7)	0 (0.0)	0.116	23 (0.7)	0 (0.0)	0.115
**Other ischemic heart disease**	59 (1.7)	58 (1.7)	1 (1.9)	0.016	59 (1.7)	0 (0.0)	0.185
**Heart failure**	27 (0.8)	26 (0.8)	1 (1.9)	0.100	27 (0.8)	0 (0.0)	0.124
**Stroke**	51 (1.4)	49 (1.4)	2 (3.8)	0.149	51 (1.5)	0 (0.0)	0.172
**Other cerebrovascular disease**	59 (1.7)	58 (1.7)	1 (1.9)	0.016	59 (1.7)	0 (0.0)	0.185
**Atrial fibrillation**	38 (1.1)	37 (1.1)	1 (1.9)	0.068	38 (1.1)	0 (0.0)	0.148
**Arrhythmia**	85 (2.4)	85 (2.5)	0 (0.0)	0.224	85 (2.4)	0 (0.0)	0.223
**Peripheral vascular disease**	32 (0.9)	32 (0.9)	0 (0.0)	0.137	32 (0.9)	0 (0.0)	0.136
**Liver disease**	103 (2.9)	99 (2.9)	4 (7.5)	0.212	103 (2.9)	0 (0.0)	0.246
**Cancer**	59 (1.7)	57 (1.6)	2 (3.8)	0.131	58 (1.7)	1 (14.3)	0.480
**Number of hospitalizations during previous year**				0.595			0.766
**0**	1644 (46.7)	1633 (47.1)	11 (20.8)		1642 (46.8)	2 (28.6)	
**1**	757 (21.5)	738 (21.3)	19 (35.8)		754 (21.5)	3 (42.9)	
**2**	511 (14.5)	501 (14.5)	10 (18.9)		510 (14.5)	1 (14.3)	
**3**	221 (6.3)	215 (6.2)	6 (11.3)		220 (6.3)	1 (14.3)	
**>3**	385 (10.9)	378 (10.9)	7 (13.2)		385 (11.0)	0 (0.0)	
**Number of hospitalizations during previous year(Psychiatry related)**				0.668			0.763

© 2023 Bosi A et al. *JAMA Network Open.*

**0**	1817 (51.6)	1805 (52.1)	12 (22.6)		1815 (51.7)	2 (28.6)	
**1**	774 (22.0)	753 (21.7)	21 (39.6)		771 (22.0)	3 (42.9)	
**2**	427 (12.1)	416 (12.0)	11 (20.8)		426 (12.1)	1 (14.3)	
**3**	207 (5.9)	202 (5.8)	5 (9.4)		206 (5.9)	1 (14.3)	
**>3**	293 (8.3)	289 (8.3)	4 (7.5)		293 (8.3)	0 (0.0)	
**Number of outpatient contacts during previous year**				0.185			0.854
**0-3**	694 (19.7)	684 (19.7)	10 (18.9)		692 (19.7)	2 (28.6)	
**3-8**	760 (21.6)	745 (21.5)	15 (28.3)		758 (21.6)	2 (28.6)	
**8-17**	936 (26.6)	925 (26.7)	11 (20.8)		936 (26.7)	0 (0.0)	
**>17**	1128 (32.1)	1111 (32.1)	17 (32.1)		1125 (32.0)	3 (42.9)	
**Number of outpatient contacts during previous year(Psychiatry related)**				0.290			0.790
**0-3**	1093 (31.1)	1073 (31.0)	20 (37.7)		1091 (31.1)	2 (28.6)	
**3-8**	757 (21.5)	745 (21.5)	12 (22.6)		755 (21.5)	2 (28.6)	
**8-17**	760 (21.6)	754 (21.8)	6 (11.3)		760 (21.6)	0 (0.0)	
**>17**	908 (25.8)	893 (25.8)	15 (28.3)		905 (25.8)	3 (42.9)	
**Lamotrigine**	1083 (30.8)	1074 (31.0)	9 (17.0)	0.333	1081 (30.8)	2 (28.6)	0.049
**Carbamazipine**	40 (1.1)	39 (1.1)	1 (1.9)	0.063	40 (1.1)	0 (0.0)	0.152
**First generation antipsychotic drugs**	511 (14.5)	500 (14.4)	11 (20.8)	0.167	508 (14.5)	3 (42.9)	0.661
**Second generation antipsychotic drugs**	2067 (58.8)	2035 (58.7)	32 (60.4)	0.034	2063 (58.8)	4 (57.1)	0.033
**Other mood stabilizers**	552 (15.7)	542 (15.6)	10 (18.9)	0.085	551 (15.7)	1 (14.3)	0.039
**Antidepressants**	2575 (73.2)	2545 (73.4)	30 (56.6)	0.359	2568 (73.1)	7 (100.0)	0.857
**Attention-deficit/hyperactivity disorder medication**	241 (6.9)	238 (6.9)	3 (5.7)	0.050	240 (6.8)	1 (14.3)	0.244
**Drugs used in addictive disorders**	214 (6.1)	211 (6.1)	3 (5.7)	0.018	214 (6.1)	0 (0.0)	0.360
**Opioids and pain medications**	624 (17.7)	613 (17.7)	11 (20.8)	0.078	622 (17.7)	2 (28.6)	0.260
**Beta blockers**	364 (10.3)	356 (10.3)	8 (15.1)	0.145	364 (10.4)	0 (0.0)	0.481
**Calcium-channel blockers**	154 (4.4)	151 (4.4)	3 (5.7)	0.060	154 (4.4)	0 (0.0)	0.303
**Diuretics**	148 (4.2)	142 (4.1)	6 (11.3)	0.273	148 (4.2)	0 (0.0)	0.297
**ACEi/ARB**	273 (7.8)	263 (7.6)	10 (18.9)	0.338	271 (7.7)	2 (28.6)	0.562
**Lipid lowering drug**	232 (6.6)	224 (6.5)	8 (15.1)	0.281	231 (6.6)	1 (14.3)	0.254

© 2023 Bosi A et al. *JAMA Network Open.*

**NSAID**	709 (20.2)	701 (20.2)	8 (15.1)	0.135	708 (20.2)	1 (14.3)	0.156
**Antiepileptic drugs**	426 (12.1)	420 (12.1)	6 (11.3)	0.025	426 (12.1)	0 (0.0)	0.526
**Total Medications**				0.**336**			0.585
**0-5**	2840 (80.7)	2803 (80.9)	37 (69.8)		2836 (80.8)	4 (57.1)	
**6-10**	629 (17.9)	613 (17.7)	16 (30.2)		626 (17.8)	3 (42.9)	
**>11**	49 (1.4)	49 (1.4)	0 (0.0)		49 (1.4)	0 (0.0)	
**Education**				0.159			
**Compulsory school**	461 (13.1)	453 (13.1)	8 (15.1)				0.578
**Secondary school**	1437 (40.8)	1414 (40.8)	23 (43.4)		461 (13.1)	0 (0.0)	
**University**	1590 (45.2)	1568 (45.3)	22 (41.5)		1434 (40.8)	3 (42.9)	
**Missing**	30 (0.9)	30 (0.9)	0 (0.0)		1586 (45.2)	4 (57.1)	

© 2023 Bosi A et al. *JAMA Network Open.*

eFigure 1. Study Design Diagram

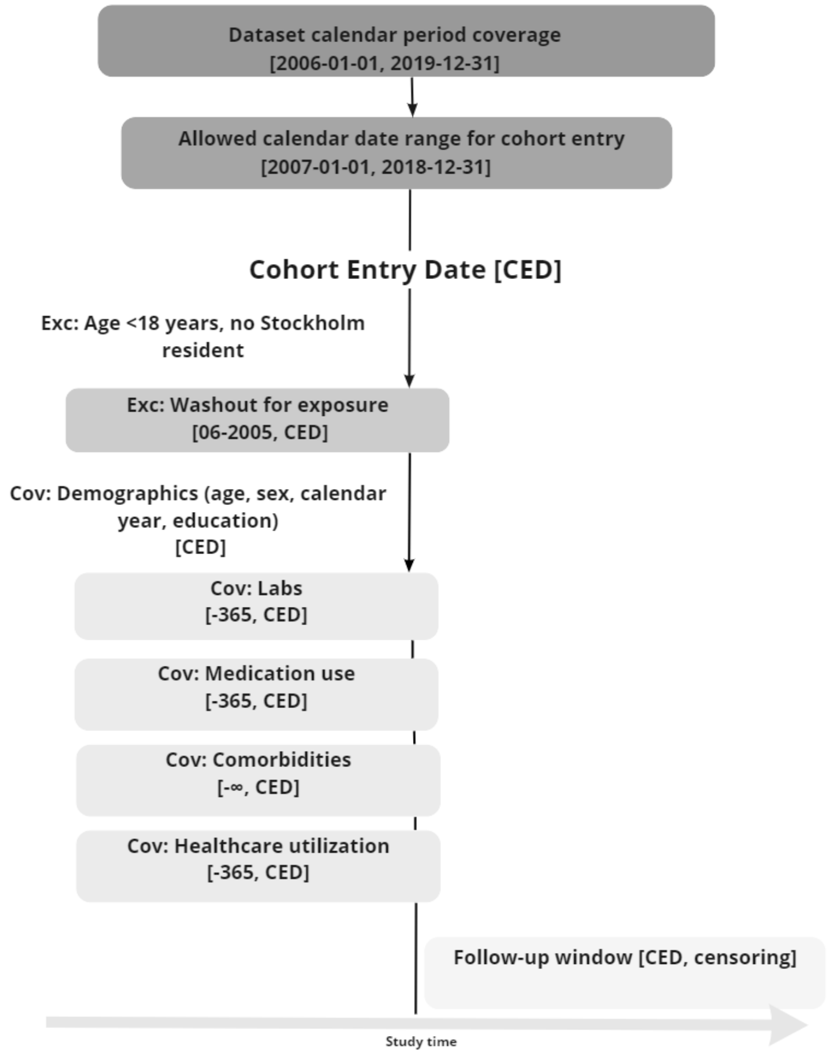

Exc excluded; Cov covariates. The date of June 2005 for washout for exposure to lithium or valproate corresponds to the date of establishment of the nationwide Prescribed Drug Register

© 2023 Bosi A et al. *JAMA Network Open.*

eFigure 2. Flowchart of Study Inclusion

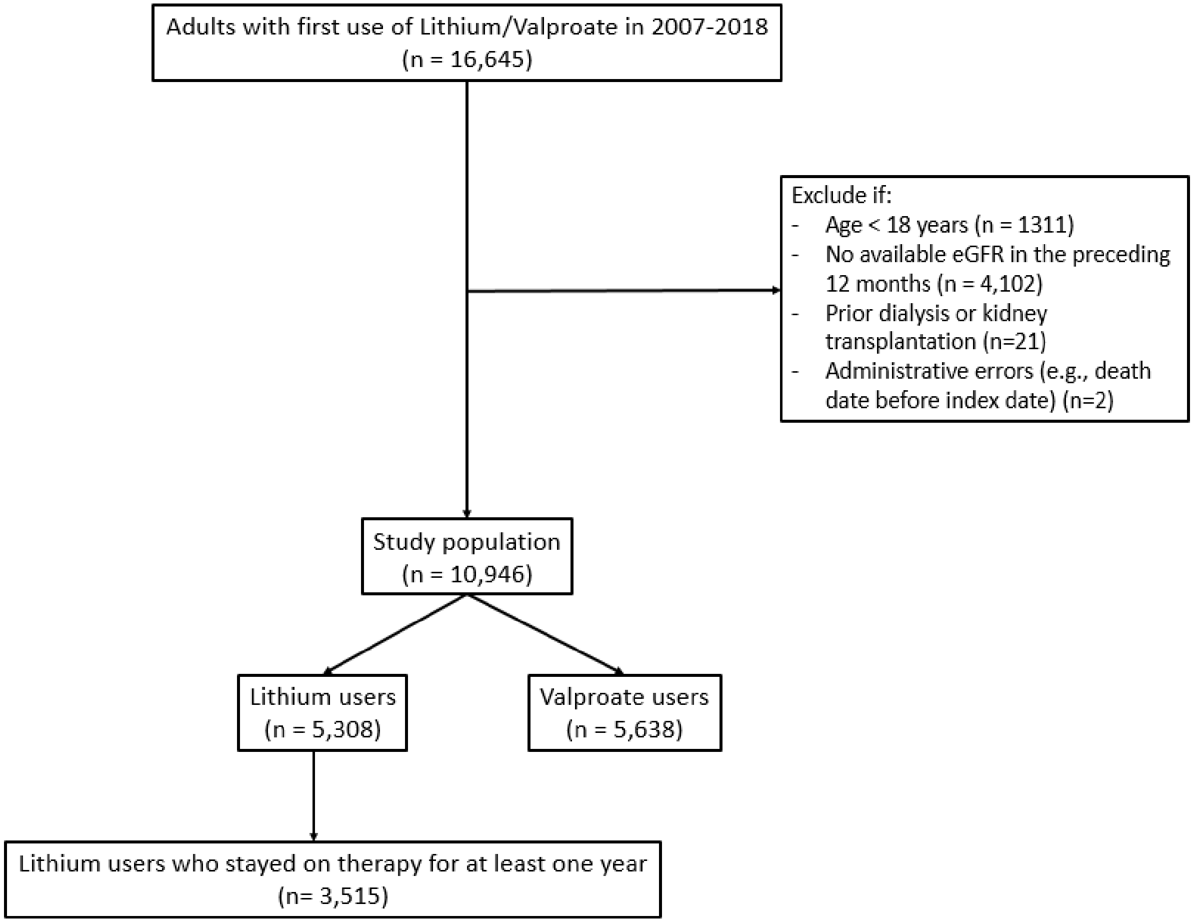

© 2023 Bosi A et al. *JAMA Network Open.*

eFigure 3. Pattern of Lithium and Valproate Prescriptions Over Time

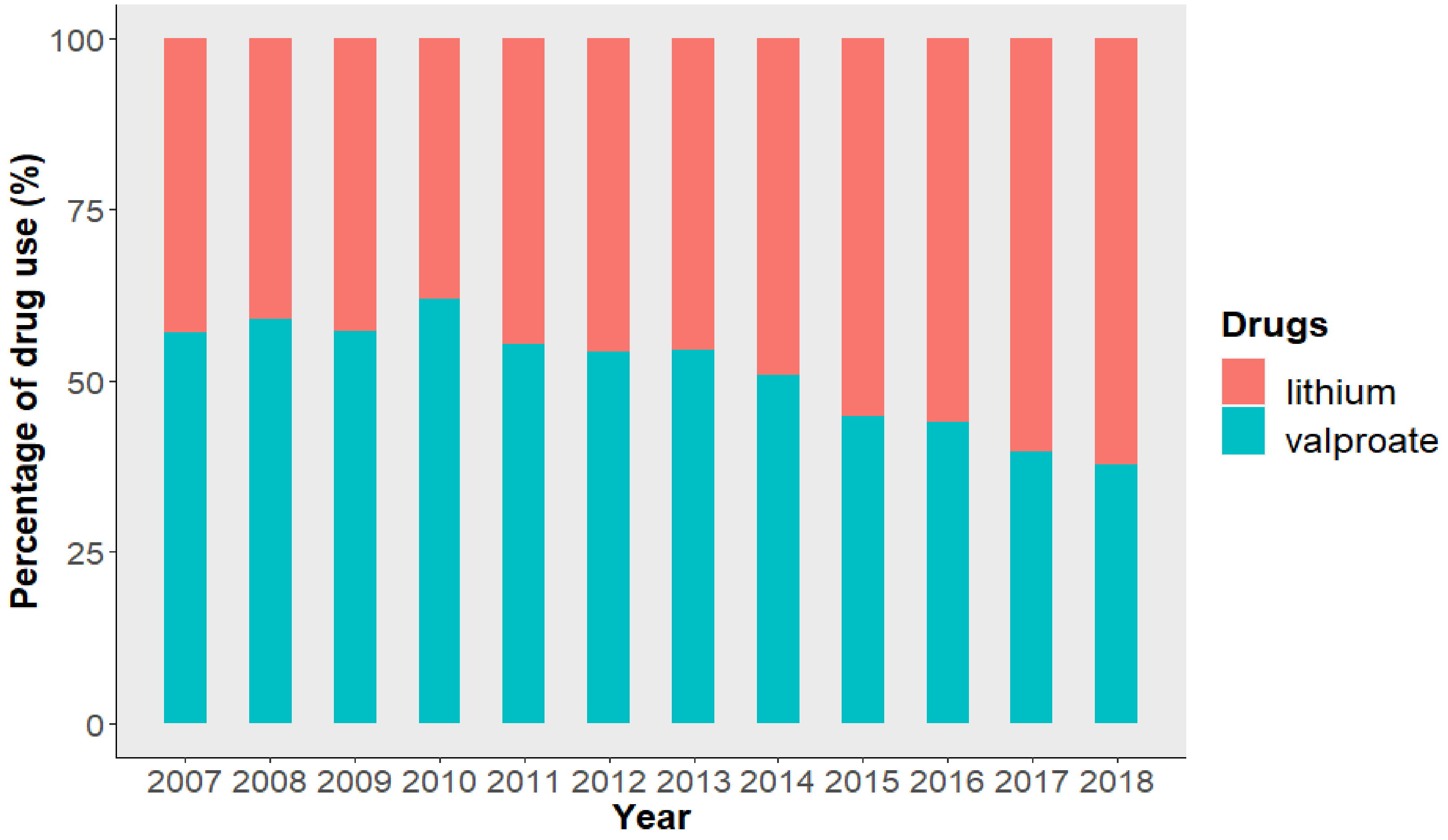

© 2023 Bosi A et al. *JAMA Network Open.*

eFigure 4. Distribution of the Time Elapsed Between First and Last Lithium Dispensation (Panel A) and Valproate (Panel B)

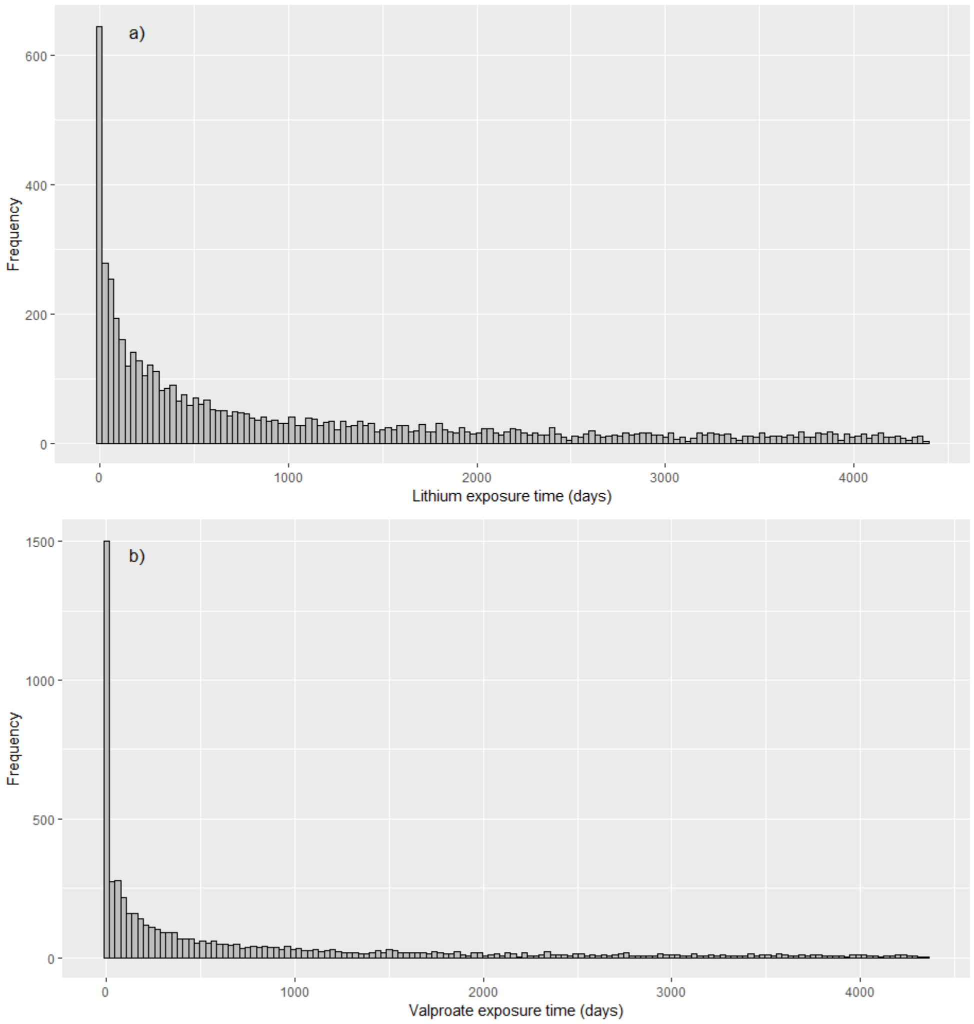

The Y axis shows the number (frequency) of lithium or valproate dispenses at pharmacies across days since therapy initiation (X axis).

© 2023 Bosi A et al. *JAMA Network Open.*

eFigure 5. Distribution of Routine Lithium Measurements Among 3518 Individuals Who Stayed on Lithium Therapy for at Least One Year

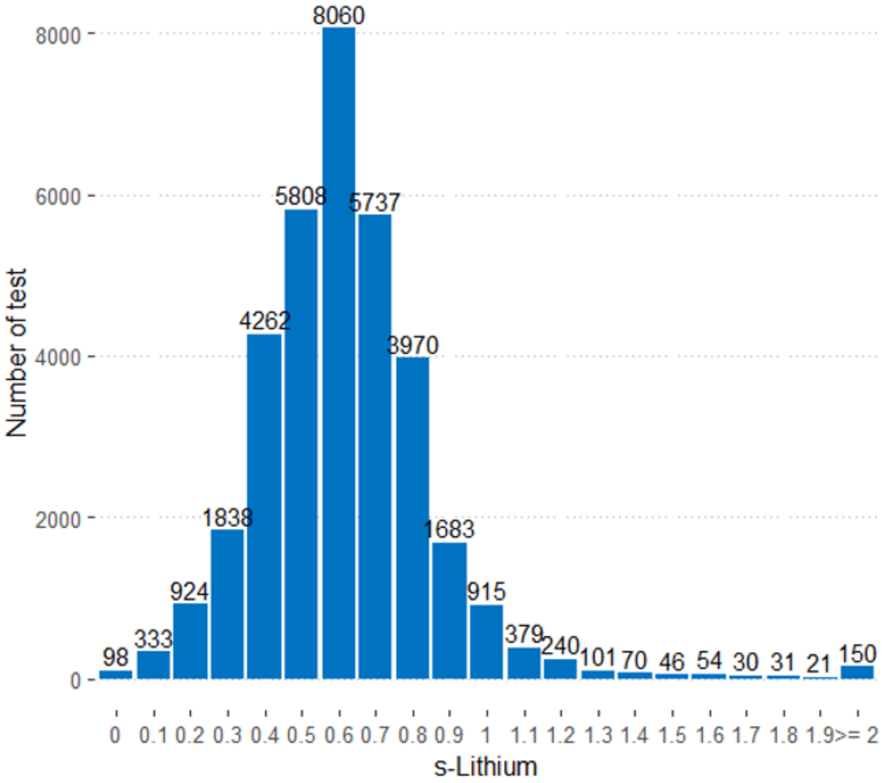

	**Overall**	**s-Li >0.8 mmol/L**	**s-Li >0.9 mmol/L**	**s-Li >0.9 mmol/L**
**Number of s-Li measurements (proportion)**	35443 (100)	3740 (11)	2045 (6)	1125 (3)
**Number of individuals (proportion)**	3518 (100)	1062 (30)	721 (21)	457 (13)

© 2023 Bosi A et al. *JAMA Network Open.*

eReferences

1. Zee, J., et al., *Using All Longitudinal Data to Define Time to Specified Percentages of Estimated GFR Decline: A Simulation Study.* Am J Kidney Dis, 2019. **73**(1): p. 82-89.
2. Kellum, J.A., et al., *Kidney Disease: Improving Global Outcomes (KDIGO) Acute Kidney Injury Work Group: KDIGO 2012 clinical practice guideline for acute kidney injury* Kidney Int Suppl. , 2012. **2**: p. 1-138.

© 2023 Bosi A et al. *JAMA Network Open.*
