# Supplementary material for: Absolute and Relative Risks of Kidney Outcomes Associated With Lithium vs Valproate Use in Sweden
Source: JAMA Netw Open. 2023 Jul 7;6(7):e2322056. doi: 10.1001/jamanetworkopen.2023.22056 (PMC10329212; doi:10.1001/jamanetworkopen.2023.22056)
Supplement: Supplement 2. — Data Sharing Statement [file jamanetwopen-e2322056-s002.pdf]

Data Sharing Statement

Bosi. Absolute and Relative Risks of Kidney Outcomes Associated With Lithium vs Valproate Use in Sweden. *JAMA Netw Open*. Published July 07, 2023.
doi:10.1001/jamanetworkopen.2023.22056

Data
Data available: No

Additional Information
Explanation for why data not available: The data cannot be made public due to GDPR regulations. However, researchers may engage in collaborative projects that are done in agreement with GDPR and regulations in Sweden at KI. Interested parties may send enquiries to Dr. Carrero (juan.jesus.carrero@ki.se).
